# Tramadol Administered Intravenously Either as a Bolus or a Slow Injection in Pain Management of Romifidine-Sedated Calves Undergoing Umbilical Hernia Repair

**DOI:** 10.3390/ani13071145

**Published:** 2023-03-24

**Authors:** Giovanna L. Costa, Fabio Leonardi, Claudia Interlandi, Patrizia Licata, Ignacio Lizarraga, Francesco Macrì, Daniele Macrì, Vincenzo Ferrantelli, Filippo Spadola

**Affiliations:** 1 Department of Veterinary Sciences, University of Messina, Via Palatucci Annunziata, 98168 Messina, Italy; 2 Department of Veterinary Science, University of Parma, Via del Taglio 10, 43126 Parma, Italy; 3 AgResearch Limited, Grasslands Research Centre, Palmerston North 4442, New Zealand; 4 Zooprophylactic Institute, Via Gino Marinuzzi 3, 90100 Palermo, Italy

**Keywords:** calves, umbilical hernia, romifidine, tramadol, slow infusion, pain

## Abstract

**Simple Summary:**

Due to the anatomical and physiological characteristics of cattle, many surgical and diagnostic procedures are often performed in cattle using standing sedation and local blocks combined with mild physical restraint. However, some procedures (e.g., abdominal surgery) are not suitable candidates for standing surgeries and are performed more easily and more safely with general anesthesia. The severity and clinical relevance of ruminants pain is probably often underestimated. The aim of the present study is to evaluate the effect of tramadol administered intravenously either as a bolus or a slow injection in romifidine-sedated calves undergoing umbilical hernia repair. Intramuscular administration of romifidine followed by tramadol either as an IV bolus or an IV slow injection provided suitable myorelaxation, deep sedation and adequate analgesia to perform umbilical hernia repair.

**Abstract:**

Umbilical hernias in calves occur with relative frequency. Most abdominal surgeries can be performed in cattle using standing sedation and local blocks. Romifidine is widely used in calves, alone or in combination with opioids. Tramadol administered as an intravenous slow injection provided better analgesia than an IV bolus in cows. The aim of the present study was to compare the response to surgical stimulus, and sedative effects of tramadol administered intravenously either as a bolus or a slow injection in romifidinesedated calves. Twenty Frisian calves undergoing umbilical hernia repair received romifidine (0.08 mg/kg IM; time 0) followed by tramadol (1 mg/kg IV) 5 min later either as a bolus (*n* = 10, B group) or a slow injection over 10 min (*n* = 10, SI group). Surgical area was infiltrated with lidocaine (4 mg/kg). Heart rate (HR), respiratory rate (RR), systolic, dyastolic and mean arterial pressure (SAP, DAP, MAP), sedation scores and response to surgical stimulus were recorded for up to 55 min. After the calves recovered a standing position, postoperative pain scores were assessed for up to 50 min. Sedation scores were significantly higher in the SI group than in the B group at 55 min (*p* < 0.05). HR, RR, SAP and response to surgical stimulus were significantly higher in the B group than in the SI group (*p* < 0.05). No significant differences were recorded in postoperative pain scores between groups (*p* > 0.05). Romifidine IM followed by intravenous tramadol, as a bolus or slow injection and local infiltration with lidocaine provided adequate sedation and analgesia in calves undergoing umbilical hernia repair.

## 1. Introduction

Due to the anatomical and physiological characteristics of cattle, many surgical and diagnostic procedures are often performed in cattle using standing sedation and local blocks combined with mild restraint [1,2]. However, ventral abdominal surgery (e.g., umbilical or inguinal hernia repair) is usually performed more easily and more safely with general anesthesia [3,4].

The list of authorized medicines for production animals (e.g., sheep, goat, and cow) is very limited both in the EU and worldwide. Nevertheless, it is a real need to have new drugs to promote good practice, animal welfare, and personnel safety. Therefore, sedatives and analgesics outside this list have been tested and used.

The α_2_-adrenoceptor agonists (α_2_-AA) xylazine, detomidine, medetomidine and romifidine are commonly used in cattle to induce reliable dose-dependent sedation and analgesia [5,6,7,8,9,10]. Stressors such as environmental and climatic factors may affect the sedation induced by α_2_-AA in cattle. Stressed animals may require larger doses to achieve a good level of sedation, and α_2_-AA could have also a delayed onset of action; furthermore, recovery may be prolonged [2,6,11,12,13,14]. It is important to underline that α_2_-AA differ from each other in selectivity for α_2_-adrenoceptors and, consequently, also in potency, duration of action and side effects [15]. Consequently, the temperament of the subject, and the α2-adrenoceptor agonist and its dose can influence the duration and the depth of sedation [2,11,12]. Additional dose of romifidine could cause significant side effects, since the α2-AA induce a short period of hypertension combined with reflex bradycardia followed by prolonged hypotension. Although there is a biphasic response with vasoconstriction and vasodilation, the degree of hypotension in awake animals is minimal. α2-AA may also induce bradypnea, hypoxia, hypercapnia, and an increase in urine output [2,8,15,16]. Atipamezole and yohimbine are α_2_-adrenoceptor antagonists that counteract these side effects but also reduce the depth of sedation and the degree of analgesia [8].

To reduce the α_2_-AA dose and their side effects, it is advisable to administer α_2_-AA combined with opioids because of their synergistic effects. This combination is widely used to provide sedation and analgesia in veterinary practice [9,10,17,18]. Tramadol is a multimodal analgesic, consisting of 50% (+)- and 50% (−)-enantiomer. Both enantiomers are weak agonists of the µ-opioid receptors, and the (+)-enantiomer inhibits the reuptake of serotonin more potently than that of noradrenaline [19]. Tramadol has been previously used in various domestic animals to induce analgesia with minimal respiratory, haemodynamic and gastrointestinal side effects [9,20,21,22]. It was administered alone or in combination with α_2_-AA to provide analgesia and sedation [9]. Its analgesic efficacy is related to the production of active metabolites dependent on hepatic function. This metabolism varies among species. Unfortunately, there are few information about analgesic efficacy of tramadol, alone or combined with α_2_-AA, in cattle [10,23].

The aims of the present study are to evaluate the effects of tramadol administered intravenously either as a bolus or a slow injection in romifidine-sedated calves undergoing umbilical hernia repair.

## 2. Materials and Methods

### 2.1. Animals

This study was performed in accordance with the Legislative Decree n. 26 of 4th March 2014 under Italian Animal Welfare Legislation and was approved by the Institutional Ethics Committee for animal welfare of the University of Messina, protocol number 027/2018. Procedures were performed following national (Italian law D.M. 116192) and international (EU Directive 2010/63/EU and USA Public Health Service Policy on Humane Care and Use of Laboratory Animals) regulations on the care and use of laboratory animals. Twenty Frisian calves from various local farms were enrolled in the study. The animals underwent surgery for umbilical hernia repair at their farms. Prior to the patients’ enrolment in the study, the owners provided informed consent.

### 2.2. Treatment Administration

A prospective, block-assigned, operator-blinded clinical trial was carried out at the farm of origin for each calf. Feed and water were withheld 6 h before surgery. On the day of surgery, the animals were weighed (OCS300, Zoo Piro, Cruto, Calabria, Italy) and, using an aseptic technique, a 14G × 5½″ catheter was inserted in an external jugular vein for medication and fluid administration. After a 30-min acclimation period in their housing pen, romifidine 0.08 mg/kg (Sedivet, Boehringer Ingelheim Animal Health, Padua, Italy) was administered intramuscularly (IM). After 5 min, tramadol 1 mg/kg (Altadol 5%, Formenti, Verona, Veneto, Italy) was administered intravenously (IV). Tramadol was administered as a bolus over 10 s (*n* = 10, B group) or as a slow injection over 10 min (*n* = 10, SI group) using an infusion pump (Angel, Burnaby, BC, Canada). Ringer’s lactate solution at a rate of 10 mL/kg/h was administered IV during surgery.

### 2.3. Umbilical Hernia Repair

After muscle relaxation, all calves were placed dorsally on a padded mattress and received tramadol IV. The umbilical region was aseptically prepared, and infiltrated with 4 mg/kg of 2% lidocaine (Lidocaina Cloridrato Esteve 2%, Ecuphar Italia S.r.l., Milan, Lombardy, Italy). Then, an open herniorrhaphy was performed [24,25,26]. An elliptical skin incision was made and, if present, adhesions of the parietal peritoneum with the skin were freed using both blunt and sharp dissection. Abdominal organs were repositioned, and the hernial ring was exposed and freshened. The linea alba was closed using 2-0 chromic catgut with an interrupted horizontal mattress suture pattern. An autologous flap was created using the remaining hernial sac. The subcutaneous tissues were closed using 2-0 chromic catgut with a simple interrupted suture. Excessive skin was removed, and the skin was closed using 2-0 nylon with a simple interrupted suture. The herniorrhaphies were carried out by two experienced surgeons working together.

### 2.4. Measurement of Physiological Parameters

Respiratory rate (RR) was determined by visual observation of thoracic excursions in one minute. Heart rate (HR), systolic, diastolic and mean arterial pressure (SAP, DAP, MAP) was measured using a multiparameter monitor (EDAN Instruments Italy, Naples, Campania, Italy arterial pressure was measured using an oscillometric method (EDAN) placing a blood pressure cuff, approximately 30–40% of the circumference of the tail, at the base of the tail. These parameters were recorded just before romifidine administration (T0, baseline values) and at 5 (T5), 10 (T10), 15 (T15), 25 (T25), 35 (T35), 45 (T45) and 55 (T55) minutes after romifidine administration.

### 2.5. Assessment of Sedation and Response to Surgical Stimulus

Quality of sedation (sedation score) was assessed at T0, T5, T10, T15, T25, T35, T45 and T55. A simple descriptive scale of 0 to 3 was used as follows: 0—no sedation, calf is alert with normal posture; 1—mild sedation, calf is in sternal recumbency and can be easily restrained in lateral recumbency; 2—moderate sedation, calf can be placed in dorsal recumbency with poor muscle relaxation; 3—marked sedation, calf can be placed in dorsal recumbency with good muscle relaxation. Scores were assigned by three trained independent observers blinded to treatment.

Response to surgical stimulus was assessed using a cumulative pain scale (CPS) [23,27,28]. A numeric score of 0 to 4 was assigned based on the percentage variation in RR, HR, and SAP from T0 (baseline values) at T10 (beginning of surgery), T15, T25, T35, T45 and T55 (end of surgery) as follows: 0 = variation ≤ 0%; 1 = variation ≤ 10%; 2 = variation > 10% but ≤20%; 3 = variation > 20% but ≤30%; 4 = variation > 30%. The sum of the scores obtained for each parameter was response to surgical stimulus.

Postoperative pain was assessed using a 10-point numerical rating scale at 20 (RT20), 30 (RT30), 40 (RT40) and 50 (RT50) minutes after calves stood [29]. An operator performed a slight compression on the surgical site by palpating with his dominant hand. Animal reaction was assessed using a numeric score of 0 to 10 as follows: 0—no reaction (no pain); 1–3—vocalisation (mild pain); 4—the calf turned its head towards stimulated area (moderate pain); 5–7—the calf turned its head towards stimulated area trying to escape (severe pain); 8–10—the calf tried to kick with pelvic limbs (very severe pain). Three trained independent observers, blinded to treatment, assessed postoperative pain.

When response to surgical stimulus was ≥10, surgical area was infiltrated with 2 mg/kg of 2% lidocaine, and the calf received sprayed intraperitoneal 2% lidocaine (2 mg/kg) as rescue analgesia. When the postoperative pain score was ≥4, the calf received 3.3 mg/kg flunixin meglumine IV (Finadyne, Schering-Plough Animal Health, Oss, The Netherlands) as rescue analgesia [30].

### 2.6. Statistical Analysis

Statistical analysis was performed by use of conventional statistical software (GraphPad Prism version 8.2.1; GraphPad Software Inc., La Jolla, CA, USA, and SPSS version 15.0; SPSS Inc., Chicago, IL, USA). Data were analysed for normality using the Shapiro-Wilk test and reported as mean ± SD or median (range) as appropriate. Demographic data were analysed using a two-tailed *t*-test, a two-tailed Mann-Whitney test or a Fisher exact t’s test as appropriate. Inter-observer agreement for quality of sedation and postoperative pain separately by treatment were analysed using the Kendall’s coefficient of concordance *W*. At each measured time, scores for quality of sedation and postoperative pain scores from the three observers were averaged. Within treatment, HR, RR, SAP, DAP, MAP and sedation differences were analysed with a Friedman test followed by a Dunn’s multiple comparison test. Differences in HR, RR, SAP, DAP, MAP, sedation, response to surgical stimulus, and postoperative pain between treatment were compared by a Mann-Whitney test at individual time points with Bonferroni correction for multiple comparisons. Values of *p* ≤ 0.05 were considered significant for all analysis. Sample size was calculated using Sample Size Calculator software, confidence level was 80%, margin of error was ±5%, population proportion was 50%, population size was 20.

## 3. Results

### 3.1. Animals and Surgery

In the present study twenty calves, with umbilical hernia, were referred, and visit, in two months, The number of subjects selected was representative of the population since the sample size was 18 subjects.

All animals completed the study, and all recoveries were uneventful. The duration of operation was approximately 45–50 min in both groups. The time from romifidine administration to standing position was approximately 150 min in both groups. All calves raised their heads about 20 min after achieving standing position.

Age, body weight, and the ratio of males to females were as follows: 59.3 ± 1.6 days (B group), 59.5 ± 1.8 days (SI group); 54.2 ± 2.3 kg (B group), 55.1 ± 2.5 kg (SI group); 0.8:0.2 (B group), 0.7:0.3 (SI group). There were no significant differences between groups regarding age (*p* = 0.8003), body weight (*p* = 0.4189) and the ratio of males to females (*p* > 0.9999).

Time from start of surgery to the animals recovering a standing position was not significantly different between groups (*p* = 0.5238), and was as follows: 245 (230–260) min (B group), 245 (240–315) min (SI group).

### 3.2. Physiological Parameters

Table 1 summarises the data about HR, RR, SAP, DAP and MAP Compared to baseline values (T0), HR was significantly decreased at T5, T25 and T45 in the B group (*p* ≤ 0.0071), and at T5, T10, T15, T45 and T55 in the SI group (*p* ≤ 0.0376). HR was significantly lower at T0, T25, T35 and T45, and significantly higher at T10 and T15 in the SI group compared to the B group (*p* < 0.05).

Compared to baseline values (T0), RR was significantly increased at T15 and T55 in the B group (*p* ≤ 0.0043), and significantly decreased at T5, T10, T35, T45 and T55 in the SI group (*p* ≤ 0.0002). RR was significantly lower at T0, and significantly higher at T10, T15, T25, T35, T45 and T55 in the B group compared to the SI group (*p* < 0.05).

Compared to baseline values (T0), SAP significantly increased at T5, T15, T35 and T55 in the B group (*p* ≤ 0.0098) and significantly decreased at T10 in the SI group (*p* = 0.0002). SAP was significantly lower at all times in the B group compared to the SI group (*p* < 0.05). DAP in the B group, compared to baseline values (T0), increased significantly at T5, T15, T25, T35, T55 (*p* ≤ 0.005); in the SI group significantly increased at T5 and T15 and decreased at T10 and T25 (*p* ≤ 0.005). Between the groups DAP in the B group was significantly lower at T0, T10, T15, T45 and increased significantly at T 35,T 55 compared to the SI group (*p* ≤ 0.005)

MAP compared to baseline (T0), significantly increased at T5, T10, T15, T25, T35, T55 in the B group (*p* ≤ 0.005); in the I group compared to T0, decreased in all time points (*p* ≤ 0.005). Between the groups, the mean pressure showed several significant variations (*p* ≤ 0.005).

### 3.3. Sedation, Response to Surgical Stimulus, and Postoperative Pain

The data are summarised in Table 1.

There was perfect interobserver agreement for quality of sedation and postoperative pain (*W* = 1).

Compared to baseline values (T0), sedation scores were significantly higher at T5, T10, T15, T25, T35 and T45 in both groups (*p* < 0.0003). At all these times, sedation scores of 3 were assigned to all calves. Sedation score was higher at T55 in the SI group (score of 2) compared to the B group (score of 1) (*p* < 0.05).

In the B group, response to surgical stimulus ranged from 2 to 9, whereas in the SI group ranged from 0 to 2 (only 3 calves had response to surgical stimulus equal to 2 at T15). Response to surgical stimulus was significantly higher at all times in the B group compared to the SI group (*p* < 0.05).

Postoperative pain scores (NRS) ranged from 0 to 4 in the B group (one calf at RT40 and 2 calves at RT50 were assigned a score of 4), and from 0 to 3 in the SI groups. There were no significant differences between groups (*p* > 0.5).

## 4. Discussion

Intramuscular romifidine administration followed by tramadol either as an IV bolus or an IV slow injection over 10 min provided satisfactory myorelaxation, deep sedation and adequate analgesia to perform umbilical hernia repair in field conditions on calves in which the umbilical region was infiltrated with lidocaine. Compared to the IV bolus administration, the IV slow injection of tramadol provided higher sedation, lower response to surgical stimulus throughout the surgical procedure, and lower postoperative pain scores. Similar results were previously reported in cows undergoing therapeutic balance of the foot [23].

It is likely that a different pharmacokinetics changed the drug distribution. We assumed that romifidine effect may have been intensified with time (from 10 to 20 min) and, consequently, cardiovascular effects may have affected the drug distribution and elimination.

HR, RR and SAP baseline values were within normal ranges in calves in both groups [31,32]. However, HR, RR SAP, DAP and MAP baseline values were 40–60% higher in calves belonging to the SI group compared to those of the B group. Recorded differences in baseline values between groups may be normal, especially in clinical trials performed in field conditions. Since calves in both groups were similarly handled, it is unlikely that preoperative procedures may have caused these differences. Nevertheless, it cannot be ruled out that calves belonging to the SI group may have been more stressed than calves belonging to the B group. It is well known that the stress may contribute to the release of catecholamines which could induce sympathomimetic effects such as tachycardia, tachypnoea and vasoconstriction. The subjects belonging to the B group were operated in winter and spring, whereas the calves belonging to the SI group were operated in summer. It is well known that environmental conditions, mainly high ambient temperature, may increase cardiorespiratory parameters of calves [33]. Consequently, it is likely that the season in which the study was performed may have affected the measured cardiorespiratory parameters [11,12,14]. Unfortunately, ambient temperature was not recorded.

Romifidine is used in Europe in daily practice for cattle but there are few scientific surveys. In this study, intramuscular romifidine administration caused a significant decrease in HR in both groups, approximately 30% in the B group and 50% in the SI group. This was to be expected because α_2_-AA induce bradycardia; an approximately 20% to 30% reduction in HR was reported in calves sedated with romifidine (0.04 mg/kg IM) [10,34]. The decrease in HR was combined with a marked increase in SAP only in the B group (approximately 50%). These findings were also expected because α_2_-AA cause vasoconstriction with resulting increase in SAP, and induce a reflex bradycardia [15]. Less evident effect in calves belonging to the SI group may be due to the higher baseline values than in calves belonging to the B group. Increases in mean arterial pressure of approximately 35% at 5 min after intravenous romifidine administration (0.05 mg/kg) were reported in sheep [15]. On the contrary, a 10% decrease in SAP was reported at 10 min after intramuscular romifidine administration (0.04 mg/kg) in calves [10]. These different effects on arterial pressure reported in previous studies may be related to the species, timing of assessments (i.e., biphasic pressor response), dose and route of administration. We have chosen to administer romifidine intramuscularly because paediatric animals have a fixed stroke volume, are more dependent on HR to maintain cardiac output, and are usually more sensitive to adverse cardiovascular effects of α_2_-AA [35].

Intramuscular romifidine administration caused also a decrease in RR in both groups, approximately 33% in the B group and 60% in the SI group. These results are consistent with those of a previous study in which a decrease of approximately 20% in RR was reported in calves receiving intramuscular romifidine (0.04 mg/kg) [34]. It is likely that respiratory depression occurred secondary to the central nervous system depression caused by α_2_- adrenoceptor stimulation

Romifidine induced safe sedation in all calves. In fact, the highest sedation scores were assigned to all animals at 5 min after romifidine administration, muscle relaxation was excellent, and all calves were easy placed in dorsal recumbency. This was to be expected because it has been previously reported that also a lower romifidine dose (0.04 mg/kg) induced recumbency for approximately 30 min [10]. On the contrary, De Rossi et al. (2005) described a moderate sedation characterised by drowsiness and drop of the head but no recumbency in healthy calves sedated with intramuscular romifidine (0.04 mg/kg) [34]. In the present study, sedation scores of 3 were maintained in all calves also for 40 min after intravenous tramadol administration. Furthermore, at 55 min after romifidine administration, sedation score was higher in the SI group than in the B group. It is likely that tramadol as a slow injection may have had longer sedative effects than tramadol administered as an intravenous bolus.

Umbilical hernia repair requires careful pain management. In the present study, pain management was based on balanced analgesia using romifidine, tramadol and regional anesthesia performed with lidocaine. The duration of analgesic effect of lidocaine is approximately 60 min after local infiltration [36]. The duration and intensity of α_2_-AA analgesic effect are dose-dependent. The analgesic effect of romifidine administered in calves (0.04 mg/kg IM) lasts approximately 90 min [10,34]. In the present study, a higher dose of romifidine was used, and it is likely that analgesic effect was stronger. The analgesic effect of tramadol has been well described in cattle. Tramadol (1 mg/kg IV) induces analgesia for at least 15 min in cows undergoing corrective claw trimming [23], and at least 60 min when combined with romifidine (0.02 mg/kg IM) in healthy calves undergoing nociceptive electrical stimulation [10]. Tramadol (4 mg/kg IV) also reduces pain-related behaviours after 30 min of a caustic paste for disbudding applied to calves [37]. To reduce romifidine dose, it is advisable to administer α2-AA combined with opioids (e.g., butorphanol) for their synergistic effects [38]. A cumulative pain scale was used to evaluate response to surgical stimulus because the percentage variations of physiological variables may objectively quantify response to noxious stimulus. Changes in physiologic parameters in response to surgical stimulus were lower in calves belonging to the SI group than those of the B group. Even though increases in cardiorespiratory parameters may be suggestive of response to noxious stimulus [39], changes in HR, RR and SAP reported in the present study preclude us from making any firm conclusions on intraoperative response to surgical stimulus. Furthermore, the decision to set a cut-off score ≥ 10 was arbitrary because there is no consensus as to when to administer rescue analgesia in anaesthetised surgical patients [40,41,42,43]. Nevertheless, similar methods of assessing response to noxious stimulus have been successfully used in dogs undergoing ovariohysterectomy [27] and in cows undergoing corrective claw trimming [23]. Therefore, we examined the percentage change from baseline values but these changes still do not indicate true response to noxious stimulus.

Early postoperative pain was assessed using a numerical rating scale which is commonly preferred to evaluate pain than other simple unidimensional scales (e.g., simple descriptive scales and visual analogue scales) [44]. Although lower scores in the SI group than in the B group were assigned to the calves’ reactions in response to surgical wound palpation, the difference was not statistically significant. Consequently, it is likely that the calves of both groups experienced similar levels of pain during the early postoperative period. Even though quality of sedation was not assessed during the postoperative period, it could be supposed that sedation of calves belonging to the SI group was longer-lasting than sedation of calves belonging to the B group. The subjective impressions of the three independent observers were that the calves were not been sedated during postoperative pain assessment because they recovered a standing position.

The present study has some limitations. The main concern is that the study is a clinical trial performed in field conditions with different environmental conditions. Moreover, the administration of tramadol with block randomization may have contributed to significant differences in baseline HR, RR, and SAP values between groups, making it difficult to explain the significance of differences between groups. Further studies are needed to evaluate the efficacy of romifidine combined with tramadol in cattle undergoing more invasive surgery.

Likewise, the differences between groups in response to noxious stimulus should be cautiously interpreted since these scores were dependent upon percentage changes from baseline for HR, RR and SAP. Pain is an emotion and cognitive response but the changes in physiologic parameters may indicate response to nociception. Therefore, if the calf is heavily sedated, its response to surgical/noxious stimulus could be evaluated with the changes of physiologic parameters.

Another limitation of the present study is that we used a modified 10-point numerical rating scale for postoperative pain assessment. Unfortunately, a validated composite scale (e.g., bovine UNESP Botucatu) would have been difficult to use because procedures have been performed in fields condition with a limited time available to assess postoperative pain. Postoperative pain assessment could have been performed by the breeders but the operators were not experienced [23,30,45]. Postoperative pain was only assessed for approximately one hour after the calves recovered a standing position. Although it would have been desirable to observe the animals for a longer period, the nature of our outpatient service prevented us from spending more time at the farms. Moreover, assessment of postoperative sedation would have allowed to understand whether or not sedation interfered with the calves’ behaviours during the postoperative period [46].

Determination of the plasma (or serum) concentrations of tramadol and its metabolites was not performed, and there are no available data for cattle in veterinary literature. Therefore, it is not possible to correlate with certainty the reported physiological, analgesic and sedative effects with the plasma concentrations of tramadol.

## 5. Conclusions

In conclusion, intramuscular romifidine administration followed by intravenous tramadol combined with local infiltration of lidocaine can be safely used in calves undergoing umbilical hernia repair in field conditions. This anesthetic management may be a feasible method to obtain deep sedation and balanced analgesia. Compared to tramadol administered as an IV bolus, tramadol administered as an IV slow injection caused lower variations in physiologic parameters in response to noxious stimulus, and lower postoperative pain scores. However, further studies are required before making any firm conclusions on intraoperative and postoperative analgesic effects.

## Figures and Tables

**Table 1:** Effect of romifidine (0.08 mg/kg IM) followed by tramadol (1 mg/kg IV) administered either as a bolus injection (B group, *n* = 10) or a slow injection over 10 min (SI group, *n* = 10) on heart rate (HR), respiratory rate (RR), systolic, diastolic, mean arterial pressure (SAP, DAP, MAP), response to surgical stimulus (cumulative pain scale; CPS), quality of sedation (simple descriptive scale; SDS), and postoperative pain (numerical rating scale; NRS) in calves undergoing umbilical hernia repair. Romifidine was administered immediately after obtaining baseline values (T0) and tramadol just after recording the T5 assessment (5 min after romifidine administration). Postoperative pain was assessed at the reported times after the calves recovered a standing position.

Parameter	Treatment	Time							
		T0 (0 min)	T5 (5 min)	T10 (10 min)	T15 (15 min)	T25 (25 min)	T35 (35 min)	T45 (45 min)	T55 (55 min)
HR (beats/min)	B	80.5 (80–84) ^†^	56.5 (54–58) *	100 (98–101) ^†^	63 (60–65) ^†^	55 (53–57) *^,†^	80 (76–82)^†^	43.5 (40–45) *^,†^	82 (80–85)
	SI	121.5 (118–125)	57.5 (54–60) *	70.5 (68–75) *	52.5 (50–56) *	89 (86–91)	91.5 (90–95)	87 (83–90) *	84.5 (81–90) *
RR (breaths/min)	B	50 (47–54) ^†^	33.5 (30–38)	43.5 (40–50) ^†^	63.5 (60–67) *^,†^	50.5 (47–54) ^†^	55 (51–58) ^†^	60 (48–64) ^†^	63 (60–66) *^,†^
	SI	80.5 (76–85)	32 (28–36) *	30.5 (28–34) *	50.5 (48–54)	35.5 (32–38)	32 (29–38) *	31.5 (28–35) *	32 (28–35) *
SAP (mmHg)	B	80.5 (76–85) ^†^	120.5 (118–124) *^,†^	80.5 (76–85) ^†^	100 (95–103) *^,†^	94.5 (90–98) ^†^	124 (121–127) *^,†^	87.5 (81–93) ^†^	118 (115–121) *^,†^
	SI	111.5 (107–115)	124.5 (121–127)	93.5 (92–97) *	121.5 (118–124)	100.5 (97–108)	105.5 (100–115)	104.5 (100–109)	102.5 (98–107)
MAP (mmHg)	B	70 (63–75) ^†^	82 (77–86) *^,†^	76.4 (73–81) *^,†^	77.8 (73–85) *^,†^	74.5 (70–80) *^,†^	86.9 (75–93) *^,†^	57.6 (55–62) *	91.8 (85–100) *^,†^
	SI	97 (95–103)	82 (77–87) *	71.5 (68–74) *	81 (79–85) *	82.5 (79–88) *	86 (81–92) *	85.8 (79–90) *	83 (74–89) *
DAP (mmHg)	B	44 (41–48) ^†^	63.3 (58–67) *	44 (40–48) ^†^	56.8 (52–65) *^,†^	56.3 (52–61) *	61.5 (57–66) *^,†^	42.8 (40–45) ^†^	64.2 (57–72) *^,†^
	SI	57.5 (55–63)	63 (59–67) *	48 (45–50) *	63.5 (61–67) *	54 (49–58) *	55.5 (51–60)	55.5 (50–60)	55 (47–60)
Response to surgical stimulus (CPS 0–12)	B			3.5 (2–4) ^†^	6 (5–7) ^†^	3 (2–3) ^†^	6 (4–7) ^†^	4 (2–5) ^†^	8 (6–9) ^†^
	SI			0 (0–0)	1 (1–2)	0 (0–0)	0 (0–1)	0 (0–1)	0 (0–0)
Sedation (SDS 0–3)	B	0 (0–0)	3 (3–3) *	3 (3–3) *	3 (3–3) *	3 (3–3) *	3 (3–3) *	3 (3–3) *	1 (1–1) ^†^
	SI	0 (0–0)	3 (3–3) *	3 (3–3) *	3 (3–3) *	3 (3–3) *	3 (3–3) *	3 (3–3) *	2 (2–2)
		***Time after the calves readopted a standing position***							
		**RT20 (20 min)**	**RT30 (30 min)**	**RT40 (40 min)**	**RT50 (50 min)**				
Postoperative pain (NRS 0–10)	B	1 (0–3)	3 (0–3)	3 (0–4)	3 (0–4)				
	SI	1 (0–2)	2 (0–3)	2 (0–3)	2 (0–3)				

* Significantly different from baseline within treatment (*p* ≤ 0.0376). ^†^ Significantly different from corresponding time point between treatments (*p* < 0.05). Within treatment differences for HR, RR, SAP and SS were analysed, separately by treatment, with a Friedman test followed by a Dunn’s multiple comparison test. Between treatment differences for HR, RR, SAP, CPS, SS and NRS were compared by a Mann-Whitney test at individual time points, with Bonferroni correction for multiple comparisons. A ≤ 0.05, B ≤ 0.01, C ≤ 0.001, D ≤ 0.0001. HR Bolus: Friedman test < 0.0001. A 0.0326 at 2 min, B 0.0071 at 20 min, D < 0.0001 at 40 min. HR Infusion: Friedman test < 0.0001. D < 0.0001 at 2 min, 5 min and 10 min, A 0.0376 at 40 min, B 0.0060 at 50 min. RR Bolus: Friedman test < 0.0001. B 0.0018 at 10 min, B 0.0043 at 50 min. RR Infusion: Friedman test < 0.0001. D 0.0001 at 2 min, D < 0.0001 at 5, 30 and 40 min, C 0.0002 at 50 min. SAP Bolus: Friedman test < 0.0001. D < 0.0001 at 2 min, B 0.0098 at 10 min, D < 0.0001 at 30 min, D 0.0001 at 50 min. SAP Infusion: Friedman test < 0.0001. C * 0.0002 at 5 min. DAP Bolus: Friedman test < 0.0001. C < 0.005 at 2, 10, 20, 30 2nd 50 min. DAP Infusion: Friedman test < 0.0001 C < 0.005 at 2, 5, 10 2nd 20 min. MAP Bolus: Friedman test < 0.0001. C < 0.005 at 2, 5, 10, 20, 30, 40 2nd 50 min. MAP Infusion: Friedman test < 0.0001 C < 0.005 at 2, 5, 10, 20, 30, 40 2nd 50 min. SDS Bolus: Friedman test < 0.0001. C < 0.0003 at 2, 5, 10, 20, 30 2nd 40 min. SDS Infusion: Friedman test < 0.0001. C < 0.0003 at 2, 5, 10, 20, 30 2nd 40 min.

## Data Availability

The data presented in this study are available on request from the corresponding author.

## References

[1] Lorena S.E.R., Luna S.P.L., Lascelles B.D.X., Corrente J.E. (2013). Attitude of Brazilian veterinarians in the recognition and treatment of pain in horses and cattle. Vet. Anaesth. Analg..

[2] Riebold T.W., Grimm K.A., Lamont L.A., Tranquilli W.J., Greene S.A., Robertson S.A. (2015). Ruminants In Veterinary Anesthesia and Analgesia.

[3] Mulon P.-Y., Desrochers A. (2005). Surgical abdomen of the calf. Vet. Clin. N. Am. Food Anim. Pract..

[4] Baird A.N. (2008). Umbilical surgery in calves. Vet. Clin. N. Am. Food Anim. Pract..

[5] Prado M.E., Streeter R.N., Mandsager R.E., Shawley R.V., Claypool P.L. (1999). Pharmacologic effects of epidural versus intramuscular administration of detomidine in cattle. Am. J. Vet. Res..

[6] Bouisset S., Saunier D., Le Blaye I., Batut V. (2002). L’association tilétamine/zolazepam romifidine dans l’anesthésie du veau. Rev. Méd. Vét..

[7] Lin H.C., Riddell M.G. (2003). Preliminary study of the effects of xylazine or detomidine with or without butorphanol for standing sedation in dairy cattle. Vet. Ther..

[8] Rioja E., Kerr C.L., Enouri S.S., McDonell W.N. (2008). Sedative and cardiopulmonary effects of medetomidine hydrochloride and xylazine hydrochloride and their reversal with atipamezole hydrochloride in calves. Am. J. Vet. Res..

[9] Costa G.L., Cristarella S., Quartuccio M., Interlandi C. (2015). Anti-nociceptive and sedative effects of romifidine, tramadol and their combination administered intravenously slowly in ponies. Vet. Anaesth. Analg..

[10] Interlandi C., Nastasi B., Morici M., Calabrò P., Costa G.L. (2017). Effects of the combination romifidine/tramadol drug administration on several physiological and behavioral variables in calves. Large Anim. Rev..

[11] Costa G.L., Nastasi B., Musicò M., Spadola F., Morici M., Cucinotta G., Interlandi C. (2017). Influence of ambient temperature and confinement on the chemical immobilization of fallow deer (Dama dama). J. Wildl. Dis..

[12] Costa G.L., Nastasi B., Musicò M., Spadola F., Morici M., Cucinotta G., Interlandi C. (2017). Reply to Arnemo and Kreeger: Influence of Ambient Temperature and Confinement on the Chemical Immobilization of Fallow Deer. J. Wild Dis..

[13] Spadola F., Costa G., Interlandi C., Musicò M. (2019). Hyaluronidase, with xylazine and ketamine, reducing immobilization time in wild cattle (Bos Taurus). Large Anim. Rev..

[14] Costa G., Musicò M., Spadola F., Oliveri M., Leonardi F., Interlandi C. (2021). Comparison of tiletamine/zolazepam combined with dexmedetomidine or xylazine for chemical immobilization of wild fallow deer (dama dama). J. Zoo Wildl. Med..

[15] Celly C.S., McDonell W.N., Young S.S., Black W.D. (1997). The comparative hypoxaemic effect of four α2 adrenoceptor agonists (xylazine, romifidine, detomidine and medetomidine) in sheep. J. Vet. Pharmacol. Ther..

[16] Kästner S.B. (2006). A2-agonists in sheep: A review. Vet. Anaesth. Analg..

[17] Lizarraga I., Castillo-Alcala F. (2015). Sedative and mechanical hypoalgesic effects of butorphanol in xylazine-premedicated donkeys. Equine Vet. J..

[18] Leonardi F., Costa G.L., Angelone M., Dubau M., Simonazzi B., Sabbioni A. (2020). Effects of intravenous romifidine, detomidine, detomidine combined with butorphanol, and xylazine on tear production in horses. Equine Vet. Educ..

[19] Interlandi C., Leonardi F., Spadola F., Costa G.L. (2021). Evaluation of the paw withdrawal latency for the comparison between tramadol and butorphanol administered locally, in the plantar surface of rat, preliminary study. PLoS ONE.

[20] Morgaz J., Navarrete R., Muñoz-Rascón P., Domínguez J.M., Fernández-Sarmiento J.A., Gómez-Villamandos R.J., Granados M.M. (2013). Postoperative analgesic effects of dexketoprofen, buprenorphine and tramadol in dogs undergoing ovariohysterectomy. Res. Vet. Sci..

[21] Monteiro B.P., Klinck M.P., Moreau M., Guillot M., Steagall P.V., Pelletier J.P., Martel-Pelletier J., Gauvin D., Del Castillo J.R., Troncy E. (2017). Analgesic efficacy of tramadol in cats with naturally occurring osteoarthritis. PLoS ONE.

[22] Costa G., Spadola F., Lentini M., Lubian E., Leonardi F. (2021). Comparison of analgesia and ataxia degree obtained between three dosages of tramadol in cattle. Large Anim. Rev..

[23] Costa G., Musicò M., Spadola F., Cortigiani S., Leonardi F., Cucinotta G., Interlandi C. (2018). Effects of tramadol slow injection vs fast bolus in the therapeutic balance of the foot in bovine. Large Anim. Rev..

[24] Spadola F., Costa G.L., Morici M., Interlandi C., Nastasi B., Musicò S.M. (2017). Autologous prosthesis for the surgery of two simultaneous hernias in a calf. Large Anim. Rev..

[25] Spadola F., Neve V.C., Interlandi C., Spadaro A., Macrì F., Iannelli N.M., Costa G.L. (2022). Hernioplasty with Peritoneal Flap for the Surgical Treatment of Umbilical Hernia in Swine. Animals.

[26] Spadola F., Neve V.C., Costa G.L., Musicò M., Spadaro A., Antoci F., Cavallo O., Cascone G. (2022). Surgical approach and etiopathogenetic considerations to the umbilical tumefactions in cattle: Case review in twenty years (2000/2020). Vet. Anim. Sci..

[27] Costa G.L., Nastasi B., Spadola F., Leonardi F., Interlandi C. (2019). Effect of levobupivacaine, administered intraperitoneally, on physiological variables and on intrasurgery and postsurgery pain in dogs undergoing ovariohysterectomy. J. Vet. Behav..

[28] Interlandi C., Di Pietro S., Costa G.L., Spadola F., Iannelli N.M., Macrì D., Ferrantelli V., Macrì F. (2022). Effects of Cisatracurium in Sevoflurane and Propofol Requirements in Dog-Undergoing-Mastectomy Surgery. Animals.

[29] Ferreira-Valente M.A., Pais-Ribeiro J.L., Jensen M.P. (2011). Validity of four pain intensity rating scales. Pain.

[30] Della Rocca G., Brondani J.T., de Oliveira F.A., Crociati M., Sylla L., Ngonput A.E., Di Salvo A., Luna S.P.L. (2017). Validation of the Italian version of the Universidade Estadual Paulista—Botucatu unidimensional composite pain scale for the assessment of postoperative pain in cattle. Vet. Anesth. Analg. Set.

[31] Piccione G., Casella S., Pennisi P., Giannetto C., Costa A., Caola G. (2010). Monitoring of physiological and blood parameters during perinatal and neonatal period in calves. Arq. Bras. Med. Vet. Zootec..

[32] Silva B.T., Henklein A., Marques R., Oliveira P.L., Leite S.B.P., Novo S.M.F., Baccili C.C., Reis J.F., Gomes V. (2016). Vital parameters of Holstein calves from birth to weaning. Rev. Bras. Med. Vet..

[33] Kovács L., Kézér F.L., Ruff F., Jurkovich V., Szenci O. (2018). Assessment of heat stress in 7 week old dairy calves with non-invasive physiological parameters in different thermal environments. PLoS ONE.

[34] DeRossi R., Miglioli L., Kassab T.A., Miguel G.L.S. (2005). Pharmacological effects of intramuscularly administration of xylazine or romifidine in calves raised on pasture. J. Anim. Vet. Adv..

[35] Grubb T., Perez J.T., Pettifer G., Grimm K.A., Lamont L.A., Tranquilli W.J. (2015). Neonatal and geriatric patients. Veterinary Anesthesia and Analgesia.

[36] Lizarraga I., Janovyak E., Beths T. (2013). Comparing lidocaine, bupivacaine and a lidocaine–bupivacaine mixture as a metacarpal block in sheep. Vet. J..

[37] Braz M., Carreira M., Carolino N., Rodrigues T., Stilwell G. (2012). Effect of rectal or intravenous tramadol on the incidence of pain-related behaviour after disbudding calves with caustic paste. Appl. Anim. Behav. Sci..

[38] Interlandi C., Calapai G., Nastasi B., Morici M., Costa G.L. (2017). Effects of Atipamezole on the Analgesic Activity of Butorphanol in Rats. J. Exot. Pet Med..

[39] Aguado D., Bustamante R., García-Sanz V., González-Blanco P., Gómez de Segura I.A. (2020). Efficacy of the Parasympathetic Tone Activity monitor to assess nociception in healthy dogs anaesthetized with propofol and sevoflurane. Vet. Anaesth. Analg..

[40] Portela D.A., Otero P.E., Briganti A., Romano M., Corletto F., Breghi G. (2013). Femoral nerve block: A novel psoas compartment lateral pre-iliac approach in dogs. Vet. Anaesth. Analg..

[41] Evangelista M.C., Silva R.A., Cardozo L.B., Kahvegian M.A.P., Rossetto T.C., Matera J.M., Fantoni D.T. (2014). Comparison of preoperative tramadol and pethidine on postoperative pain in cats undergoing ovariohysterectomy. BMC Vet. Res..

[42] Ávila Rodríguez A., Buriticá-Gaviria E., Echeverry-Bonilla D. (2016). Intraoperative analgesic effects of intratesticular lidocaine in a dog undergoing elective orchiectomy: Case report. Orinoquia.

[43] Oliva V.N.L.S., Albuquerque V.B., Floriano B.P., Meneghetti T.M., Abimussi C.J.X., Ferreira J.Z., Wagatsuma J.T., Laranjeira G.M., Santos P.S.P. (2019). Different rates of tramadol infusion for peri and postoperative analgesia in dogs undergoing orthopedic surgery. Arq. Bras. Med. Vet. Zootec..

[44] Mathews K., Kronen P.W., Lascelles D., Nolan A., Robertson S., Steagall P.V., Wright B., Yamashita K. (2014). Guidelines for recognition, assessment and treatment of pain. J. Small Anim. Pract..

[45] Lomax S., Windsor P.A. (2013). Topical anesthesia mitigates the pain of castration in beef calves. J. Anim. Sci..

[46] Reedman C.N., Duffield T.F., DeVries T.J., Lissemore K.D., Duncan I.J., Winder C.B. (2021). Randomized controlled trial assessing the effects of xylazine sedation in 2- to 6-week-old dairy calves disbudded with a cautery iron. J. Dairy Sci..

